# Additional treatment after primary conservative treatment in patients with chronic subdural hematoma—A retrospective study

**DOI:** 10.1002/brb3.3590

**Published:** 2024-07-02

**Authors:** Rahman Fakhry, Clemens M. F. Dirven, Walid Moudrous, S. Mirjam Droger, Nabil Asahaad, Christiaan de Brabander, Hester F. Lingsma, Niels A. van der Gaag, Heleen M. den Hertog, Bram Jacobs, Korné Jellema, Ruben Dammers, Dana C. Holl

**Affiliations:** ^1^ Department of Neurosurgery Erasmus Medical Center, Erasmus MC Stroke Center Rotterdam The Netherlands; ^2^ Department of Neurology Maasstad Hospital Rotterdam The Netherlands; ^3^ Department of Neurology Van Weel Bethesda Hospital Dirksland The Netherlands; ^4^ Department of Neurology Admiraal de Ruyter Hospital Goes The Netherlands; ^5^ Department of Public Health Erasmus Medical Center Rotterdam The Netherlands; ^6^ University Neurosurgical Center Holland (UNCH), Leiden University Medical Center, Haaglanden Medical Center, Haga Teaching Hospital Leiden The Netherlands; ^7^ Department of Neurology Isala Hospital Zwolle Zwolle The Netherlands; ^8^ Department of Neurology University of Groningen University Medical Center Groningen Groningen The Netherlands; ^9^ Department of Neurology Haaglanden Medical Center The Hague The Netherlands

**Keywords:** conservative, CSDH, treatment

## Abstract

**Objective:**

Chronic subdural hematoma (CSDH) is a common neurological condition and is typically treated with burr hole craniostomy. Nevertheless, conservative treatment may lead to spontaneous hematoma resolution in some patients. This study aims to describe the characteristics of patients who were treated conservatively without the eventual need for additional treatment.

**Methods:**

Data were retrospectively collected from patients who were primarily treated conservatively in three hospitals in the Netherlands from 2008 to 2018. The Primary outcome was the nonnecessity of additional treatment within 3 months after the initial CSDH diagnosis. We used univariable and multivariable logistic regression analyses to identify factors associated with not receiving additional treatment.

**Results:**

In this study, 83 patients were included and 61 patients (73%) did not receive additional treatment within 3 months. Upon first presentation, the patients had a Markwalder Grading Scale score (MGS) of 0 (*n* = 5, 6%), 1 (*n* = 43, 52%), and 2 (*n* = 35, 42%). Additional treatment was less often received by patients with smaller hematoma volumes (adjusted odds ratio [aOR] 0.78 per 10 mL; 95% confidence interval [CI] 0.64–0.92). Patients using antithrombotic medication also received less additional treatment, but this association was not significant (aOR 2.02; 95% CI 0.61–6.69).

**Conclusions:**

Three quarters of the initially conservatively treated CSDH patients do not receive additional management. Typically, these patients have smaller hematoma volumes. Further, prospective research is needed to distinguish which patients require surgical intervention and in whom primary conservative treatment suffices.

## Graphical abstract

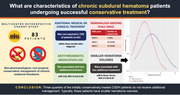

## 1 INTRODUCTION

Chronic subdural hematoma (CSDH) is a highly prevalent neurological disease with an incidence that ranges from 1.72 to 79.4 per 100,000 individuals per year (Adhiyaman et al., 2017; Asghar et al., 2002; Balser et al., 2015; Foelholm & Waltimo, 1975; Karibe et al., 2011; Kudo et al., 1992; Rauhala et al., 2019). The incidence has recently increased, which is likely due to an aging population and the increased use of antithrombotics in these patients (Adhiyaman et al., 2017; Rauhala et al., 2019).

There is currently no consensus on the optimal treatment for CSDH. In symptomatic patients, the mainstay of treatment is surgical management, most often through burr hole craniostomy (Cenic et al., 2005; Ernestus et al., 1997; Santarius et al., 2008). Nonetheless, there is still an ongoing debate on the optimal surgical management of CSDH (Solou et al., 2022). Given the older age of the majority of CSDH patients, a surgical approach carries a relatively increased risk of complications and case fatality (Lee et al., 2018; Shlobin et al., 2021). Alternative treatments, such as the use of pharmacological agents, have also been studied. Specifically, the use of dexamethasone has been studied before, as its anti‐inflammatory and antiangiogenic properties may be beneficial in resolving the hematoma (Edlmann et al., 2017; Holl et al., 2018). However, the recent Dex‐CSDH trial as well as the DECSA study have shown that treatment with dexamethasone resulted in fewer favorable outcomes and more complications compared with placebo and/or surgery (Hutchinson et al., 2020; Miah et al., 2023).

Alternatively, conservative treatment can be considered, which encompasses close monitoring of the patient without utilizing medical and/or surgical intervention(s). It has been reported that in some patients, CSDH may resolve spontaneously (Göksu et al., 2009; Juković et al., 2012). Conservative management may therefore be a viable treatment strategy in some patients diagnosed with CSDH (Kim et al., 2016), as it would also prevent these patients from undergoing surgical treatment and its associated complications. A national survey in the Netherlands found that 56% of responding neurologists and neurosurgeons had a positive experience with conservative treatment for newly diagnosed CSDH (Holl, Blaauw, et al., 2022). However, it remains unclear which individual patients, diagnosed with CSDH, may benefit from this conservative approach.

This study aims to describe the characteristics of patients who were treated conservatively and did not receive any additional medical or surgical treatment. Although dexamethasone is no longer a recommended form of treatment for CSDH, it was prescribed during the study period.

## 2 MATERIALS AND METHODS

### 2.1 Study design

This retrospective study was conducted at the neurology departments of three hospitals in the region of Rotterdam, The Netherlands: Maasstad Hospital in Rotterdam, Admiraal de Ruyter Hospital in Goes, and Van Weel Bethesda Hospital in Dirksland. Patients undergoing surgical management of CSDH were referred and transferred to the neurosurgical department of the Erasmus Medical Centre in the same region. The design of this retrospective study has previously been described (Holl, Fakhry, et al., 2022).

### 2.2 Patient population

Patients were eligible for inclusion in this study if they were diagnosed with CSDH between January 1, 2008, and December 31, 2018, and received primary conservative treatment, also known as a “wait‐and‐see” policy or as “non‐pharmacological” and/or “nonsurgical” treatment. Patients were excluded when subdural hyperdense (hemorrhagic) components were seen on the baseline head computed tomography (CT) or magnetic resonance imaging (MRI) scan comprising more than one‐third of the hematoma.

### 2.3 Treatment

For the complete inclusion period, primary conservative management was a treatment strategy in CSDH patients that was not suited for medical or surgical management. These patients were generally followed up after discharge for every 3 weeks in the first 2 months and for every 6 weeks in the remaining 4 months. In the event of patient improvement during this period, further follow‐up was discontinued. In the event of clinical deterioration after primary conservative treatment, additional medical or surgical treatment was considered.

Additionally, prior antithrombotic medication usage and possible discontinuation for each patient were carefully evaluated. The discontinuation of antithrombotic medication, along with the decision to resume antithrombotic therapy, was made on an individual basis.

### 2.4 Outcomes

The primary outcome of this study was the nonnecessity of additional treatment within 3 months post‐diagnosis. Secondary outcomes included complications, mortality, and functional status (2–10 weeks and 10–13 weeks after diagnosis).

Baseline patient characteristics were collected and included age, sex, symptoms at diagnosis, Markwalder Grading Scale (MGS) (Markwalder et al., 1981), history and time point of possible preceding head trauma, medical history, use of medication, and head CT/MRI scan parameters such as midline shift, hematoma thickness, hematoma volume, side, and hematoma type (homogeneous, laminar, separated, trabecular, or a combination of hematoma types) on the CT/MRI scan at diagnosis.

Functional outcome was difficult to measure with the modified Rankin Scale (mRS) score, since the follow‐up data, as found in the electronic patient files, were often incomplete. Therefore, the following pragmatic scale was used to determine the functional outcome: If a patient recovered well, or had only minimal residual symptoms, the overall functional status was registered as “good.” If there were still symptoms present that impact the patient's daily functioning, but no additional treatment was necessary, an overall functional status of “moderate” was entered. “Poor outcome” was used when a patient needed additional treatment. This outcome was scored up to 3 months after the diagnosis.

### 2.5 Data collection

The data collection methods of our retrospective study have previously been described. (Holl, Fakhry, et al., 2022) In summary, data were retrospectively collected from electronic medical records, registered on paper Clinical Registration Forms, and afterward processed in SPSS 25.0 (IBM Corp.). First, the diagnosis of CSDH was confirmed by a single investigator (Dana C. Holl) and, when in doubt, discussed with one of the coauthors (Ruben Dammers). Most CT/MRI data were radiologically assessed by a single investigator (Dana C. Holl). Hematoma volumes were radiologically measured, using Brainlab AG, by another single investigator (Rahman Fakhry) and randomly reviewed by a second investigator (Ruben Dammers). The 3‐month mortality was scored “unknown” if no patient data were available at 3 months. The study protocol was approved by the Medical Review Ethics Committee (Rotterdam, registration number MEC‐2019‐0710).

### 2.6 Statistical analysis

Baseline and demographic characteristics were summarized by means and standard deviations (SD) for continuous variables and numbers and percentages for categorical variables and were compared between two groups: patients receiving additional treatment within 3 months and patients receiving no additional treatment. The study employed univariable and multivariable logistic regression analysis to examine the relationship between patient characteristics (specifically, the use of antithrombotic medication and hematoma volume) and the need for additional treatment. The model included age, sex, hematoma volume, and antithrombotic medication usage as variables. Age and sex were incorporated into the regression model, since these covariates are typically regarded as fundamental contributors to the analysis. The selection of the hematoma volume variable was predetermined due to its clinical relevance and established association with additional treatment (Tian et al., 2022; Wang et al., 2022). The inclusion of antithrombotic medication as a variable was determined after careful examination of the available data. The associations were presented as adjusted odds ratios (aORs) with 95% confidence interval (CI). The data were analyzed using IBM SPSS Statistics (Version 25) and R Statistical Software.

## 3 RESULTS

**FIGURE 1:**
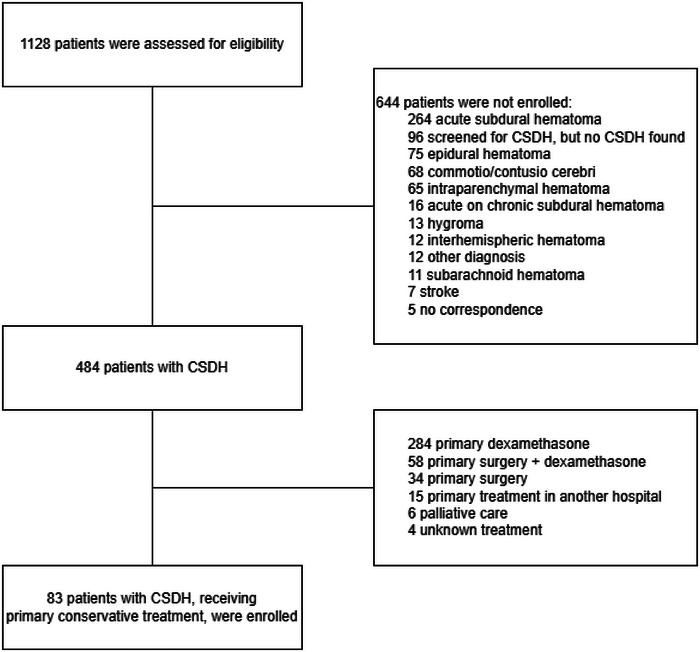
Flowchart screening for eligibility. CSDH, chronic subdural hematoma.

Between January 1, 2008, and December 31, 2018, 484 patients were diagnosed with CSDH. 83 patients (17%) initially received conservative treatment and were therefore included in the present study (Figure 1).

**FIGURE 2:**
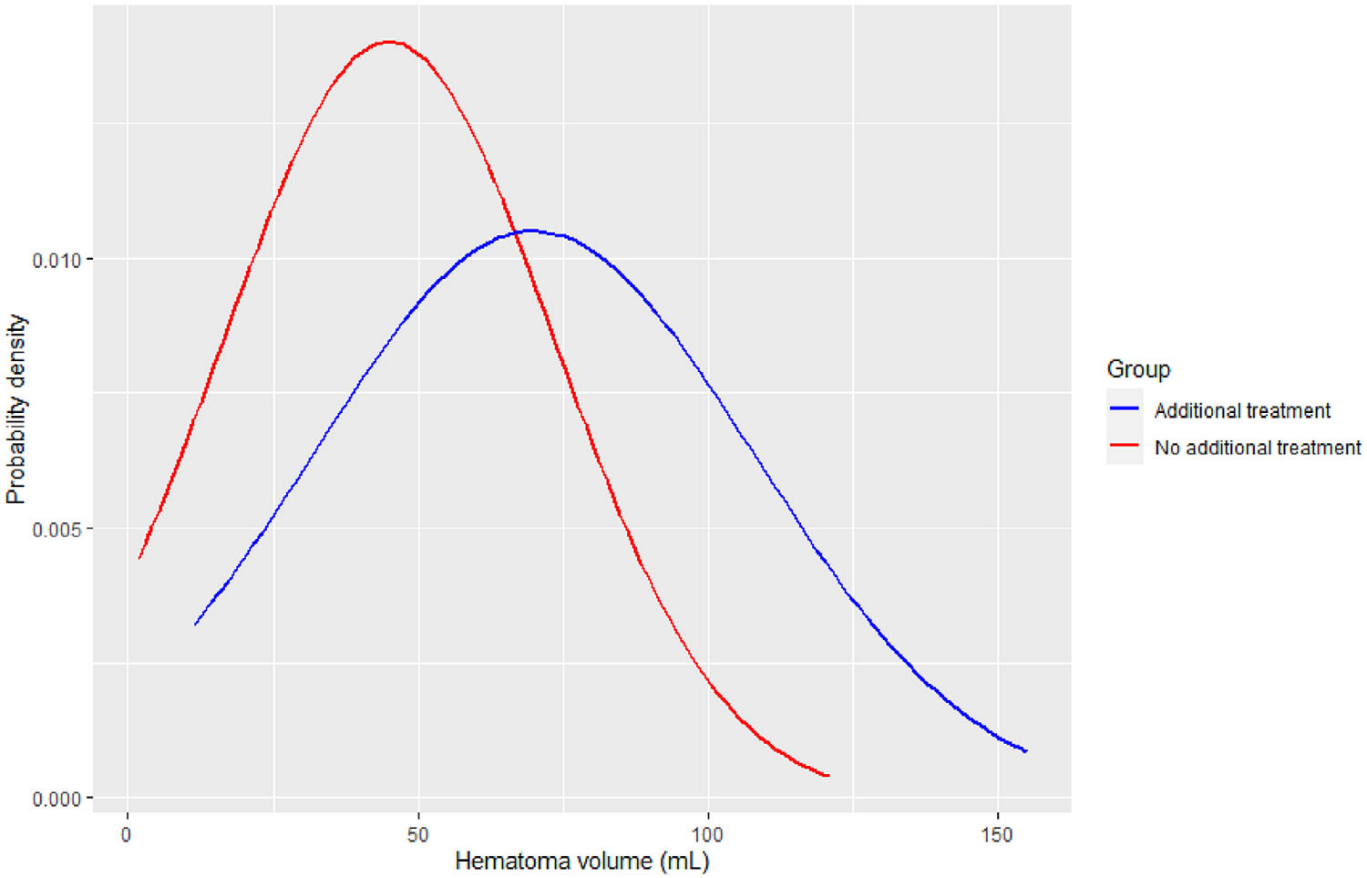
Distributions of hematoma volumes of both groups. The distribution curves of the hematoma volume variable are represented, stratified by the binary variable additional treatment. The *x*‐axis represents the hematoma volume values, indicating the range of observed volumes. The *y*‐axis represents the probability density, which indicates the relative likelihood of observing a particular hematoma volume value in each group. The red curve corresponds to the group receiving no additional treatment, while the blue curve corresponds to the group receiving additional treatment. The figure shows a higher probability of observing smaller hematoma volume values in the group receiving no additional treatment. The distributions intersect at a hematoma volume value of 66.7 mL.

The mean (SD) age at diagnosis was 78 (Solou et al., 2022) years and the majority of patients were male (*n* = 57, 69%) (Table 1). Most patients had an MGS of 1 (*n* = 43, 52%) or 2 (*n* = 35, 42%). A total of 53 patients (64%) used antithrombotics (anticoagulants and/or thrombocyte inhibitors). Baseline CT/MRI showed a bilateral CSDH in 22 patients (27%), a mean (SD) midline shift in unilateral CSDH of 3.7 mm (3.1), a hematoma thickness of <10 mm in 53 patients (64%), a mean (SD) hematoma volume of 51 mL (33), and a homogeneous hematoma type in 54 patients (65%) (Table 1; Figure 2). The hematoma volume distributions of the two groups intersect at a hematoma volume value of 66.7 mL (Figure 2).

**TABLE 1:** Characteristics of the patients at baseline.

Characteristic	Total (N = 83)	No additional treatment received (N = 61)	Additional treatment received (N = 22)
Age (years)	77.9 ± 10.6	79.0 ± 11.1	75.0 ± 8.9
Male sex, no./total no. (%)	57/83 (68.7)	43/61 (70.5)	14/22 (63.6)
Charlson comorbidity index	4.7 ± 1.9	4.9 ± 1.9	4.2 ± 1.8
Symptoms, no./total no. (%)			
Headache	36/83 (43.4)	26/61 (42.6)	10/22 (45.5)
Gait disturbance	31/83 (37.3)	21/61 (34.4)	10/22 (45.5)
Altered mental status	24/83 (28.9)	14/61 (23.0)	10/22 (45.5)
Hemiparesis	12/83 (14.5)	7/61 (11.5)	5/22 (22.7)
Speech disorder	18/83 (21.7)	11/61 (18.0)	7/22 (31.8)
Seizure(s)	5/83 (6.0)	3/61 (4.9)	2/22 (9.1)
Nausea and vomiting	10/83 (12.0)	6/61 (9.8)	4/22 (18.2)
Markwalder Grading Scale score, no./total no. (%)			
0: No neurological symptoms	5/83 (6.0)	5/61 (8.2)	0/22 (0)
1: Alert, oriented. Mild symptoms such as headache	43/83 (51.8)	32/61 (52.5)	11/22 (50.0)
2: Drowsy or disoriented with variable deficits	35/83 (42.2)	24/61 (39.3)	11/22 (50.0)
Known head trauma, no./total no. (%)	53/83 (63.9)	39/61 (63.9)	14/22 (63.6)
Days trauma to diagnosis	43.2 ± 40.2	41.9 ± 38.9	46.4 ± 44.8
Main coexisting medical conditions, no./total no. (%)			
None	4/83 (4.8)	1/61 (1.6)	3/22 (13.6)
Ischemic heart disease	13/83 (15.7)	10/61 (16.4)	3/22 (13.6)
Atrial fibrillation	22/83 (26.5)	19/61 (31.1)	3/22 (13.6)
Hypertension	36/83 (43.4)	26/61 (42.6)	10/22 (45.5)
Previous stroke	10/83 (12.0)	9/61 (14.8)	1/22 (4.5)
Diabetes	20/83 (24.1)	14/61 (23.0)	6/22 (27.3)
Statin use	25/83 (30.1)	22/61 (36.1)	3/22 (13.6)
Atorvastatin use	5/83 (6.0)	5/83 (6.0)	0/83 (0)
Any antithrombotic medication, no./total no. (%)	53/83 (63.9)	43/61 (70.5)	10/22 (45.5)
Bilateral CSDH on CT/MRI scan, no./total no. (%)	22/83 (26.5)	16/61 (26.2)	6/22 (27.3)
Midline shift in unilateral CSDH on CT/MRI scan in mm, no./total no. (%)	3.7 ± 3.1	3.0 ± 2.7	5.4 ± 3.6
<5 mm	43/61 (70.5)	35/45 (77.8)	8/16 (50.0)
5–10 mm	16/61 (26.2)	9/45 (20.0)	7/16 (43.8)
≥10 mm	2/61 (3.3)	1/45 (2.2)	1/16 (6.3)
Hematoma thickness^a^ on CT/MRI scan in mm, no./total no. (%)	9.4 ± 4.9	8.4 ± 3.7	12.1 ± 6.6
<10 mm	53/83 (63.9)	43/61 (70.5)	10/22 (45.5)
10–20 mm	27/83 (32.5)	18/61 (29.5)	9/22 (40.9)
≥20 mm	3/83 (3.6)	0/61 (0)	3/22 (13.6)
Hematoma volume^a^, ^b^ on CT/MRI scan in mL	51.4 ± 32.8	45.0 ± 28.5	69.9 ± 38.0
Hematoma type on CT scan, no./total no. (%)			
Homogeneous	54/83 (65.1)	40/61 (65.6)	14/22 (63.6)
Laminar	6/83 (7.2)	4/61 (6.6)	2/22 (9.1)
Separated	3/83 (3.6)	0/61 (0)	3/22 (13.6)
Trabecular	10/83 (12.0)	7/61 (11.5)	3/22 (13.6)
Combined	2/83 (2.4)	2/61 (3.3)	0/22 (0)
Missing^c^	8/83 (9.6)	8/61 (13.1)	0/22 (0)

Abbreviations: CSDH, chronic subdural hematoma; CT, computed tomography; MRI, magnetic resonance imaging.

^a^ Hematoma thickness and volume in bilateral CSDH: largest hematoma was included.

^b^ Hematoma volume was only found in 81 patients because of incomplete data.

^c^ Hematoma type was missing when an MRI scan was used for diagnosis.

The majority of additionally treated patients received medical treatment with dexamethasone (*n* = 15, 68%) (Table 2; Table A1 for more details). The mean (SD) from diagnosis to additional treatment was 18 (Hutchinson et al., 2020) days, with a median (min–max) of 12 (2–60) days. The most frequent complication in the group receiving no additional treatment was infection (*n* = 10, 16%). In the group receiving additional treatment, hyperglycemia (*n* = 5, 23%), infection (*n* = 3, 14%), and epileptic seizures (*n* = 2, 9%) were the most frequent complications (Table 2; full overview of complications in Table A1). “Good” functional outcome at 3 months was observed in 40 patients (66%) in the group receiving no additional treatment and in 12 patients (55%) in the group receiving additional treatment. Death within 3 months occurred in four patients (7%) in the group receiving no additional treatment and in two patients (9%) in the group receiving additional treatment (Table 2; Table A2).

**TABLE 2:** Descriptives of treatment and outcome.

Characteristic	Total (N = 83)	No additional treatment received (N = 61)	Additional treatment received (N = 22)
Type of additional treatment, no./total no. (%) Dexamethasone Surgery Dexamethasone + surgery	n/a n/a n/a	n/a n/a n/a	15/22 (68.2) 4/22 (18.2) 3/22 (13.6)
Admission—total days mean ± SD	6.1 ± 8.9	3.4 ± 5.6	13.6 ± 11.8
Diagnosis to additional treatment, days mean (SD) median (min–max)	n/a n/a	n/a n/a	17.9 (16.4) 12 (2–60)
Cessation of anticoagulants/ antithrombotics Total patients, no./total no. (%) Total days mean ± SD	39/53 (73.6) 67.5 ± 65.8	30/39 (77.0)^a^ 68.8 ± 72.1	9/10 (90.0) 62.2 ± 38.4
Complications within 3 months, no./total no. (%)			
Infection	13/83 (15.7)	10/61 (16.4)	3/22 (13.6)
Hyperglycemia	7/83 (8.4)	2/61 (3.3)	5/22 (22.7)
Pulmonary embolism	1/83 (1.2)	0/61 (0)	1/22 (4.5)
Thrombotic events (other than lung)	1/83 (1.2)	1/61 (1.6)	0/22 (0)
Epilepsy	2/83 (2.4)	0/61 (0)	2/22 (9.1)
Last known overall functional status within 3 months, no./total no. (%)			
Good	52/83 (62.7)	40/61 (65.6)	12/22 (54.5)
Moderate	5/83 (6.0)	2/61 (3.3)	3/22 (13.6)
Poor	3/83 (3.6)	1/61 (1.6)	2/22 (9.1)
Death	6/83 (7.2)	4/61 (6.6)	2/22 (9.1)
Unknown	17/83 (20.5)	14/61 (23.0)	3/22 (13.6)

^a^ Cessation of anticoagulants/antithrombotics was missing in four patients that did not receive additional treatment.

Of the 83 patients, 81 were included in regression analysis. Two patients had no available CT data for the calculation of the hematoma volumes and were therefore excluded. Additional treatment was less often performed in patients with smaller hematoma volumes (aOR 0.78 per 10 mL; 95% CI 0.64–0.92) and less often in patients with prior use of antithrombotic medication (aOR 2.02; 95% CI0.61–6.69) (Table 3).

**TABLE 3:** Factors associated with additional treatment.

	Univariable	Multivariable
Covariates	OR (95% CI)	aOR (95% CI)
Age	1.03 (0.98–1.08)	1.02 (0.97–1.08)
Sex	0.64 (0.23–1.88)	0.42 (0.12–1.40)
Hematoma volume^a^	0.79 (0.67–0.93)	0.78 (0.64–0.92)
Prior antithrombotic medication use	2.78 (1.00–7.90)	2.02 (0.61–6.69)

Abbreviations: aOR, adjusted odds ratio; CI, confidence interval; OR, odds ratio.

^a^ Per 10 mL change in hematoma volume.

## 4 DISCUSSION

This multicenter, retrospective study of 83 patients diagnosed with CSDH, primarily treated conservatively, aimed to assess the characteristics of patients eventually not receiving any additional medical or surgical treatment. About three quarters of these primarily conservatively treated patients were not additionally treated with corticosteroids and/or surgery. A smaller hematoma volume and the use of antithrombotic medication were associated with a decreased likelihood of receiving additional treatment. Age and sex did not seem to be associated with the likelihood of receiving additional treatment.

Previous studies have already shown that larger hematoma volumes are associated with a higher risk of additional treatment in patients with CSDH (Tian et al., 2022; Wang et al., 2022). One study (Wang et al., 2022) found that larger hematoma volumes were an independent risk factor for surgery in conservatively managed patients. The authors set a cutoff value at 68.5 mL, with smaller hematoma volumes having a higher chance of successful conservative management. This is comparable to the value of 66.7 mL found in this study (Figure 2). Another study (Tian et al., 2022) also reported that larger hematoma volumes at admission were associated with lower rates of self‐absorption in patients with CSDH. This finding supports the use of hematoma volume as a prognostic factor of the likelihood of self‐absorption in patients with CSDH. We found that the use of antithrombotic medication was associated with a lower likelihood of receiving additional treatment, although not significant. However, it remains uncertain whether this association is due to an actual reduction in the need for additional treatment or due to physicians' reluctance to perform such interventions. Physicians may have been hesitant to perform surgery in patients with a strong indication for antithrombotics. Contrary to our current views, at that time, it was thought that dexamethasone could be used as an alternative noninvasive treatment. Therefore, in patients with a strong indication for antithrombotics, physicians might have chosen to administer dexamethasone in order to spare patients the risks associated with surgery. Further research is needed to clarify the underlying reasons for the observed association between antithrombotic medication and a lower likelihood of receiving additional treatment.

The results of this study have potential implications for clinical practice, as they suggest that hematoma volume should be considered as a prognostic factor of the need for additional treatment in patients with CSDH. Additionally, the findings indicate that the prior use of antithrombotic medication should not be the sole determinant of treatment indication in patients with CSDH.

This study shows that there are a limited number of predictors of successful conservative treatment of CSDH. Future research should focus on prospective studies and prediction models by focusing on long‐term functional patient outcomes after conservative treatment. The Dutch chronic Subdural Hematoma Research group (DSHR) is currently working on a national, prospective database including all adult CSDH patients (Dutch chronic Subdural Hematoma Research group, 2018).

This study has several limitations. First of all, given the retrospective nature of the study, there is a higher risk of information bias. This could have led to missing information on complications, standard measurements, and baseline characteristics. The mRS could also not be determined. Alternatively, a pragmatic functional outcome scale was designed to still offer information about functional patient outcome. Another limitation of this retrospective study is that the determination of the need for additional treatment was based on the judgment of the treating physician(s). This could introduce confounding bias, as physicians may be influenced by certain factors, such as large hematoma volumes and the use of antithrombotic medication, leading to overtreatment. Lastly, the sample size in this study was relatively small, which limited the number of covariates that could be included in the multivariable logistic regression analysis. Despite this limitation, the study was able to identify one potential predictor of successful conservative treatment of CSDH, which can serve as a basis for further research in this area.

## 5 CONCLUSIONS

In conclusion, this study found that three quarters of patients diagnosed with CSDH and receiving primarily conservative treatment did not receive additional management. A smaller hematoma volume and a history of antithrombotic medication were associated with a decreased likelihood of receiving additional treatment. Further, prospective research is needed to distinguish the individual patient who can benefit from surgery from the individual patient who can safely be treated conservatively.

## AUTHOR CONTRIBUTIONS

**Rahman Fakhry**: Investigation; Visualization; Formal Analysis; Writing ‐ Original Draft. **Clemens M. F. Dirven**: Conceptualization; Supervision; Funding acquisition. **Walid Moudrous**: Resources; Writing ‐ Review & Editing. **S. Mirjam Droger**: Resources; Writing ‐ Review & Editing. **Nabil Asahaad**: Resources; Writing ‐ Review & Editing. **Christiaan de Brabander**: Resources; Writing ‐ Review & Editing. **Hester F. Lingsma**: Conceptualization; Methodology; Formal Analysis; Writing ‐ Review & Editing; Funding acquisition. **Niels A. van der Gaag**: Writing ‐ Review & Editing. **Heleen M. den Hertog**: Writing ‐ Review & Editing. **Bram Jacobs**: Writing ‐ Review & Editing. **Korné Jellema**: Writing ‐ Review & Editing. **Ruben Dammers**: Conceptualization; Investigation; Supervision; Writing ‐ Original Draft; Funding acquisition. **Dana C. Holl**: Conceptualization; Methodology; Validation; Investigation; Supervision; Writing ‐ Original Draft; Funding acquisition; Project administration.

## CONFLICT OF INTEREST STATEMENT

The authors declare no conflicts of interest.

### PEER REVIEW

The peer review history for this article is available at https://publons.com/publon/10.1002/brb3.3590

## Supporting information

Table A.1. Characteristics of the patients receiving additional treatment
Table A.2. Causes of death.

## Data Availability

The data that support the findings of this study are available from the corresponding author upon reasonable request.
