# Supplementary material for: Additional treatment after primary conservative treatment in patients with chronic subdural hematoma—A retrospective study
Source: Brain Behav. 2024 Jul 2;14(7):e3590. doi: 10.1002/brb3.3590 (PMC11219291; doi:10.1002/brb3.3590)
Supplement: Supplementary file 1 — Table A.1. Characteristics of the patients receiving additional treatment Table A.2. Causes of death. [file BRB3-14-e3590-s001.docx]

**Supplementary Material**

**Table A.1. Characteristics of the patients receiving additional treatment**

| Patients | Type of additional treatment | Total amount of dexamethasone in mg | Total days dexamethasone | Complications within 3 months | Complications before/after additional treatment |
| --- | --- | --- | --- | --- | --- |
| 1 | Dexamethasone | 64.5 | 16 | No complications | - |
| 2 | Dexamethasone | 89.5 | 39 | Epilepsy | After |
| 3 | Dexamethasone | 127.0 | 12 | Falling | Before |
| 4 | Dexamethasone | 45.0 | 13 | Saddle pulmonary embolism | After |
| 5 | Dexamethasone | 412.5 | 54 | Infection | After |
| 6 | Dexamethasone | 544.0 | 113 | Mood disorder | Before |
| 7 | Dexamethasone | 109.5 | 43 | Hyperglycemia | Before |
| 8 | Dexamethasone | 142.0 | 17 | Stomach ache | After |
| 9 | Dexamethasone | 158.5 | 22 | Falling | Before |
| 10 | Dexamethasone | 232.5 | 25 | Infection | After |
| 11 | Dexamethasone | 105.0 | 36 | Missing | - |
| 12 | Dexamethasone | 102.5 | 22 | No complications | - |
| 13 | Dexamethasone | 47.5 | 28 | Hyperglycemia  Deep vein thrombosis | After  After |
| 14 | Dexamethasone | 49.5 | 32 | No complications | - |
| 15 | Dexamethasone | 59.0 | 23 | Vegetative complaints | After |
| 16 | Dexamethasone + surgery | 215.0 | 20 | Hyperglycemia | After |
| 17 | Dexamethasone + surgery | 215.0 | 20 | Hyperglycemia | After |
| 18 | Dexamethasone + surgery | 62.5 | 11 | Hyperglycemia | Before |
| 19 | Surgery | n/a | n/a | No complications | - |
| 20 | Surgery | n/a | n/a | Delirium  Epilepsy  Falling | After  After  After |
| 21 | Surgery | n/a | n/a | No complications | - |
| 22 | Surgery | n/a | n/a | Delirium  Infection (pneumonia) | Before  Before |

**Table A.2. Causes of death**

| Patients | Additional treatment | Type of additional treatment | Cause of death | Days from diagnosis until death |
| --- | --- | --- | --- | --- |
| 1 | No | n/a | Pneumonia/sepsis | n/a |
| 2 | No | n/a | Pneumonia | n/a |
| 3 | No | n/a | Unknown | n/a |
| 4 | No | n/a | Terminal renal insufficiency | n/a |
| 5 | Yes | Dexamethasone | Unknown | 48 |
| 6 | Yes | Dexamethasone | Saddle pulmonary embolism | 34 |
